# Extracting gene expression patterns and identifying co-expressed genes from microarray data reveals biologically responsive processes

**DOI:** 10.1186/1471-2105-8-427

**Published:** 2007-11-02

**Authors:** Jeff W Chou, Tong Zhou, William K Kaufmann, Richard S Paules, Pierre R Bushel

**Affiliations:** 1 Microarray Group, National Institute of Environmental Health Sciences, Research Triangle Park, North Carolina, USA; 2 Department of Pathology and Laboratory Medicine, Center for Environmental Health and Susceptibility, and Lineberger Comprehensive Cancer Center, University of North Carolina at Chapel Hill, Chapel Hill, North Carolina, USA

## Abstract

**Background:**

A common observation in the analysis of gene expression data is that many genes display similarity in their expression patterns and therefore appear to be co-regulated. However, the variation associated with microarray data and the complexity of the experimental designs make the acquisition of co-expressed genes a challenge. We developed a novel method for Extracting microarray gene expression Patterns and Identifying co-expressed Genes, designated as EPIG. The approach utilizes the underlying structure of gene expression data to extract patterns and identify co-expressed genes that are responsive to experimental conditions.

**Results:**

Through evaluation of the correlations among profiles, the magnitude of variation in gene expression profiles, and profile signal-to-noise ratio's, EPIG extracts a set of patterns representing co-expressed genes. The method is shown to work well with a simulated data set and microarray data obtained from time-series studies of dauer recovery and L1 starvation in C. elegans and after ultraviolet (UV) or ionizing radiation (IR)-induced DNA damage in diploid human fibroblasts. With the simulated data set, EPIG extracted the appropriate number of patterns which were more stable and homogeneous than the set of patterns that were determined using the CLICK or CAST clustering algorithms. However, CLICK performed better than EPIG and CAST with respect to the average correlation between clusters/patterns of the simulated data. With real biological data, EPIG extracted more dauer-specific patterns than CLICK. Furthermore, analysis of the IR/UV data revealed 18 unique patterns and 2661 genes out of approximately 17,000 that were identified as significantly expressed and categorized to the patterns by EPIG. The time-dependent patterns displayed similar and dissimilar responses between IR and UV treatments. Gene Ontology analysis applied to each pattern-related subset of co-expressed genes revealed underlying biological processes affected by IR- and/or UV- induced DNA damage.

**Conclusion:**

EPIG competed with CLICK and performed better than CAST in extracting patterns from simulated data. EPIG extracted more biological informative patterns and co-expressed genes from both C. elegans and IR/UV-treated human fibroblasts. Using Gene Ontology analysis of the genes in the patterns extracted by EPIG, several key biological categories related to p53-dependent cell cycle control were revealed from the IR/UV data. Among them were mitotic cell cycle, DNA replication, DNA repair, cell cycle checkpoint, and G_0_-like status transition. EPIG can be applied to data sets from a variety of experimental designs.

## Background

A common observation in the analysis of gene expression is that many genes are co-regulated[1,2]. When genes are co-regulated under various biological conditions, the corresponding expression profiles may display relative similarity, or co-expression. To identify these co-expressed genes, various cluster and factor analysis methods have been applied to microarray datasets. Among the most popular unsupervised clustering methods used for the analysis of gene expression data are hierarchical clustering [2], *K*-means [3], self-organizing maps (SOM) [4], Clustering Affinity Search Technique (CAST) [5], partitioning around medoids (PAM) [6] and CLICK[7,8]. These clustering methods, e.g. SOM and *K*-means, provide a number of cluster centroids to which genes with similar expression profiles are closely situated. However, it is well-known that the number of clusters or varying the starting seed in these cluster methods can produce very different results. In addition, these clustering methods are vulnerable to the presence of "scattered" genes [9] and can lack robustness when there is little spatial separation between the clusters [10]. Numerous alternative methods have been developed to improve the utility of *K*-means and SOM for clustering gene expression data, such as clustering based on pre-defined sets of gene expression profiles [11,12]. Supervised clustering methods such as those utilizing predefined patterns [11,12], require a priori knowledge of the underlying pattern(s) in the data and may allow exclusion of unknown but biologically meaningful patterns.

CLICK, a clustering algorithm based on graph theory connectivity, a probabilistic framework and fundamental statistics, does not rely on assumptions or prior knowledge about the clusters or their structure, yet identifies tight and highly similar groups (kernels) that are likely to belong to the same true cluster [7]. Overall homogeneity (the degree of similarity of elements in the same cluster), and separation of elements in clusters from each other, are two criteria that CLICK relies on to evaluate the quality of the cluster structure. These measures are similar to the intra-compactness and inter-separation distances indices that are used to evaluate the validity of clustering solutions [13].

Among factor analysis methods are independent component analysis (ICA) [14,15] and partial least squares (PLS) [16]. ICA and PLS approaches for analysis of gene expression data generally provide biologically meaningful patterns among the top listed components which consist of a large number of co-expressed genes. However, if a perturbation to a biological system stimulates a relatively large number of co-expressed patterns or when a pattern contains only a small number of co-expressed genes, many of them may not be revealed by these methods. We present a novel profile-based method for Extracting microarray gene expression Patterns and Identifying co-expressed Genes, designated as EPIG. Through analysis of correlations among profiles, the magnitude of variation in gene expression within profiles, and evaluation of the profile signal-to-noise ratios, EPIG extracts a set of patterns representing co-expressed genes. Using a simulated data set, we compared EPIG to CLICK and CAST to evaluate the generation of the appropriate number of patterns. In addition, we applied EPIG and CLICK to a C. elegans dauer recovery and L1 starvation time course gene expression data set [17] to compare the ability of the algorithms to extract patterns related to either dauer-specific recovery or L1 starvation. Finally, we applied EPIG to a combined UV and IR treated time-series data set. Through Gene Ontology analysis of the co-expressed genes, enriched categories provided hints to underlying co-expression, biological processes, and the molecular function of the genes.

## Results

To evaluate the ability of EPIG to extract patterns from gene expression data, we used both EPIG and CLICK to analyze a simulated data set, a publicly available dauer recovery and L1 starvation gene expression data set from Caenorhaditis elegans [17,18] and the UV and IR treated fibroblast lines gene expression data. CLICK was selected as the comparator because, as similar to EPIG, the method does not rely on any assumptions or prior knowledge about the clusters or their structure unlike that of SOMs or *K*-means. The extraction of patterns and categorization of co-expressed genes by EPIG is analogous to clustering the genes (all or the differentially expressed ones) by CLICK. In other words, a pattern in EPIG is equivalent to the pattern extracted from the centroid of a cluster generated by CLICK.

### Comparison of CLICK and EPIG using simulated data

**Figure 1:**
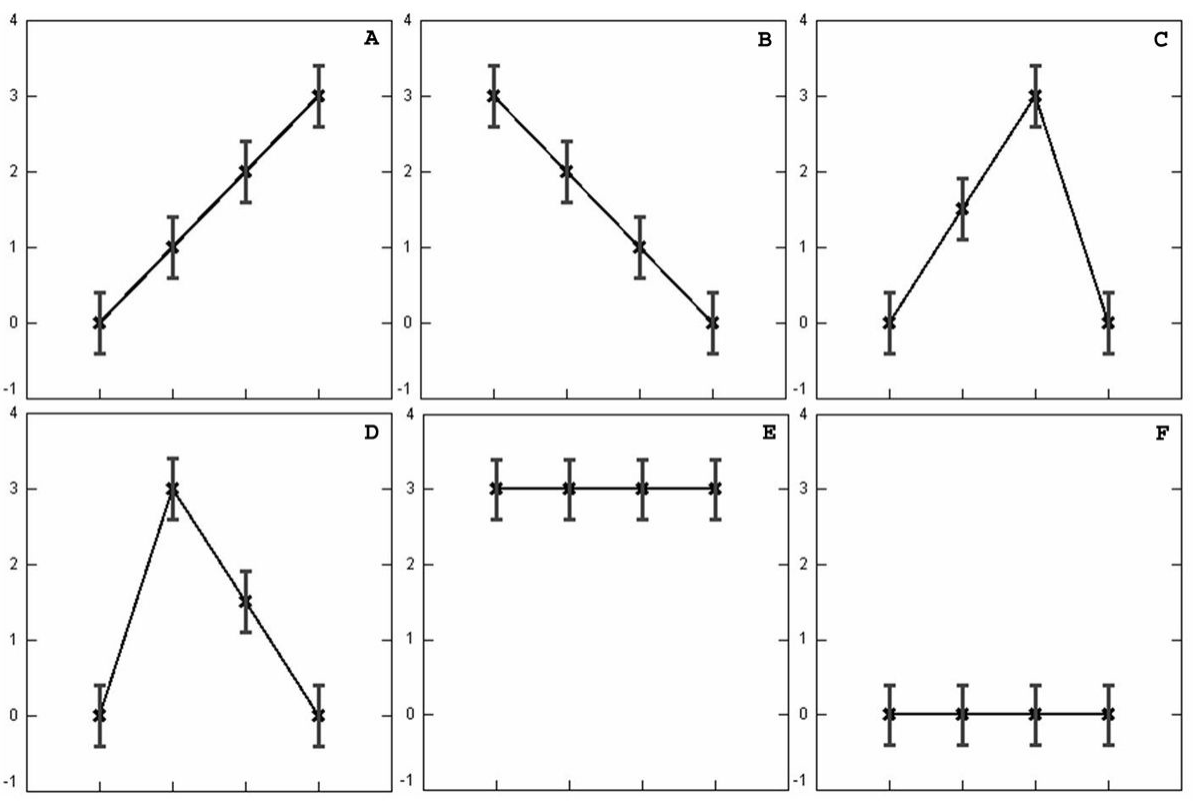
**Six probability distribution profiles**. Plot of the six probability distribution profiles in terms of mean values and standard deviations given in Table 1. In each of the figures from (A) to (F), there are four data points marked as crosses. The four data points from left to right correspond to inter-group 1 to 4, respectively. The labels of the vertical axis indicate the mean values of the data points. The vertical bars are the standard deviation of 0.4 to each of the mean values.

**Figure 2:**
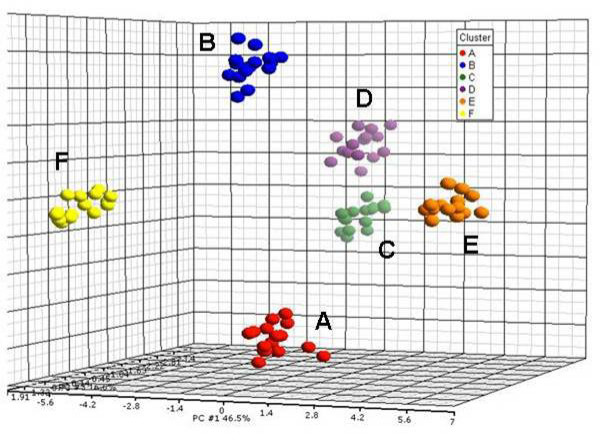
**Plot of first three components of a PCA using 90 simulated profiles**. The six clusters, from A to F, labelled in different colors correspond to the distributions from A to F in table 1. Each of the clusters consists of 15 profiles generated. 84.3% of the variability in the data was captured by the first 3 principal components (PCs). The x-axis is PC1, the y-axis PC2 and the z-axis PC3.

**Figure 3:**
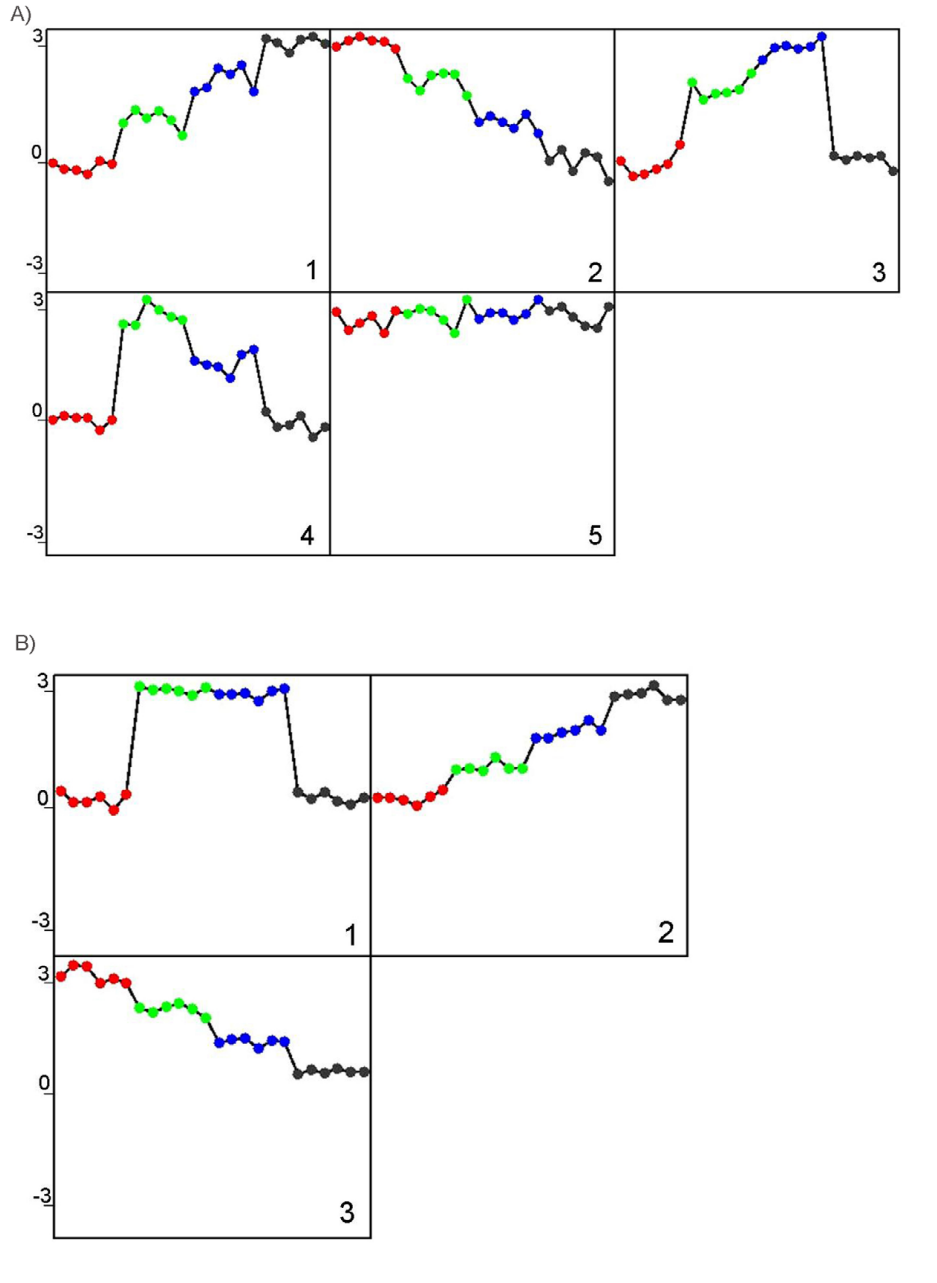
**Patterns of the simulated data extracted by EPIG and CLICK**. The four inter-groups (red, green, blue and black) from left to right in each pattern correspond to the inter-groups from 1 to 4 shown in Table 1. A) The patterns extracted by EPIG are labelled from 1 to 5 correspond to the distributions A to E, respectively. All profiles were categorized to their respective pattern. B) The pattern extracted by CLICK from Cluster 1 with 32 profiles assigned to it appears to have emerged from both distributions C and D in Table 1. The patterns for Clusters 2 and 3 correspond to distributions A and B in Table 1. The two clusters have 16 and 15 profiles assigned respectively.

Table 1 lists the mean value distributions used in a simulation of data where the standard deviation was set to be constant at 0.4. Figure 1 displays the mean values of the six probability distributions for generating the data, where Figure 1(A) and 1(B) are monotonically up and down, respectively. Figure 1(C) and 1(D) start and end at zero but peak at the third and second data points respectively. Figure 1(E) and 1(F) are flat at 3 and 0, respectively. The mean values equal to zero in Figure 1(F) reflect a flat response analogous to real gene expression data in which a number of genes may be not responsive to a given series of treatments. Normal deviates were drawn at random to generate 15 profiles for each of the six distributions. Figure 2 is a principal component analysis (PCA) of the simulated data, where the six distinct clusters of the profiles are distributed in 3-dimensional space. When the simulated data was analyzed by EPIG and CLICK for comparison, EPIG extracted 5 patterns from the data (see Figure 3A) corresponding to profile probability distributions depicted in Figures 1(A) to 1(E) and assigned all of the profiles to their proper patterns (15 profiles each) with 100% accuracy, except for the pattern from the distribution of the data shown in Figure 1(F) and its corresponding and uncategorized profiles (the ones generated to best represent a nonresponsive gene expression pattern). On the other hand, CLICK with the default homogeneity setting, only returned 3 clusters with the patterns of the centroids as shown in Figure 3B from the data omitting the clusters from the distribution of the data shown in Figure 1(E) which had all inter-group mean values at 3. CLICK merged the profiles from the patterns of the distributions of the data of Figure 1(C) and 1(D) together, despite the peaks at the distinct data points in the patterns. In addition, CLICK assigned 32 profiles (two of them from the distribution E in Table 1 or Figure 1(E)) to its Cluster 1 and 16 profiles (one of them also from the distribution E) to its Cluster 2. We also varied the homogeneity setting. With homogeneity settings at 0.83 or 0.84, CLICK generated the highest overall average homogeneity within the patterns and produced essentially the same three clusters as were produced using the default setting (data not shown).

**Table 1:** Intra-group means of simulated profiles where the standard deviation was set at 0.4.

Intra-group means	Inter- group 1	Inter- group 2	Inter- group 3	Inter- group 4	Number of profiles generated
Distribution A	0	1	2	3	15
Distribution B	3	2	1	0	15
Distribution C	0	1.5	3	0	15
Distribution D	0	3	1.5	0	15
Distribution E	3	3	3	3	15
Distribution F	0	0	0	0	15

### Analysis of dauer recovery and L1 starvation gene expression from Caenorhabditis elegans

A data set consisting of gene expression profiles from a C. elegans dauer recovery and L1 starvation time course study was obtained to compare EPIG's and CLICK's ability to extract patterns/clusters the data for identification of co-expressed genes from a publicly available microarray data set. The experimental design and other details of the study can be obtained from Wang et al [17]. Applied to the data which included all the genes, EPIG extracted 18 patterns of gene expression and identified 1597 co-expressed genes (see extracted patterns in Figure S1 and heat map of the 1597 genes in Figure S2 in Additional file 1). The numbers of genes categorized to each of the patterns varied from 15 to 263 (see Table S1 in Additional file 1). On the other hand, CLICK generated only 6 clusters of genes from the data (1644 selected genes, based on SNR (>3) and magnitude of expression (>0.5), including all 1597 genes identified by EPIG) as shown by the patterns of the centroids in Figure S3 in Additional file 1. If we applied CLICK to the whole gene data set as what was done for EPIG, CLICK produced over 60 clusters (data not shown).

In dauer-specific processes, there were four groups of genes corresponding to the dauer recovery: transient, early, climbing and late [17]. Table 2 lists the dauer recovery-specific gene expression patterns from both EPIG and CLICK corresponding to the four groups of co-expressed genes along with the dauer enriched state. There were no clusters generated by CLICK which contained genes with expression patterns related to either early or climbing states. However, there were four patterns which corresponded to the transient, late and dauer enriched states. On the other hand, the patterns of the genes extracted by EPIG corresponded to each of these dauer recovery response groups. For example, there were four patterns (Patterns 2, 3, 10 and 11 in Figure S1 in Additional file 1) in which expression levels of all of the genes decreased from early to late corresponding to the dauer transient state. However, these four patterns differed slightly from one another. For instance, Patterns 2 and 10 have no change at the start, then the expression of the genes gradually decreases to be substantially down-regulated at the end. However, their corresponding L1 starvation expression levels were either down-regulated (in the case of Pattern 2) or not changed (in the case of Pattern 10) across all the time points. Conversely, Patterns 3 and 11 reflect no change at all time points in L1 starvation, but the response of the genes to dauer recovery were either up-regulated at the start, then gradually decreased to no change at end (in the case of Pattern 3) or had no change at the start, were up-regulated early, then gradually decreased to no change at end (in the case of Pattern 11). Similarly, one may relate EPIG's Patterns 6, 7 and 9 to the dauer recovery early state and Pattern 14 to the climbing state. There were no clusters generated from CLICK that contained patterns of the centroids corresponding to these two dauer-specific states (see Figure S3 in Additional file 1). The patterns from EPIG and CLICK showing similar responses between dauer recovery and L1 starvation were not listed in Table 2.

**Table 2:** Dauer recovery-specific gene expression profiles.

	EPIG			CLICK		
	pattern number	Dauer recovery	L1 starvation	pattern number	Dauer recovery	L1 starvation

Transient	2	No change at start, gradually decrease, to significantly down regulated at end	Down regulated at all time points	1	From up regulated to no change	Down regulated
	3	Up regulated at start, gradually decrease to no change at end	No change at all time points			
	10	No change at start, gradually decrease, to significantly down regulated at end	No change at start, then, stay minimally down regulated	6	No change at start, gradually decrease, to significantly down regulated at end	Down regulated
	11	No change at start, jump to significantly up regulated after the start, gradually decrease to no change at end	No change at all time points			
Early	6	Down regulated at 0 h. from up regulated to minimally up regulated	No or minimal change			
	7	Up regulated at 0 h, peak at 5 h, then to minimally up regulated	Minimally down regulated			
	9	From no change to down regulated	No change or Minimally down regulated			
Climbing	14	Down regulated at 0 h, climbing to peak at 3 to 5 h	No or minimal change			
Late	12	Up regulated at late time points	Up regulated at late time points	4	Up regulated at late time points	Up regulated at late time points
Dauer enriched	4	Down regulated	From up regulated to no change	2	Down regulated	From up regulated to no change

Those patterns not listed below possess similar responses between dauer recovery and L1 starvation except the late responses.

### Expression patterns and co-expressed genes in response to UV- and/or IR-induced DNA damage

**Figure 4:**
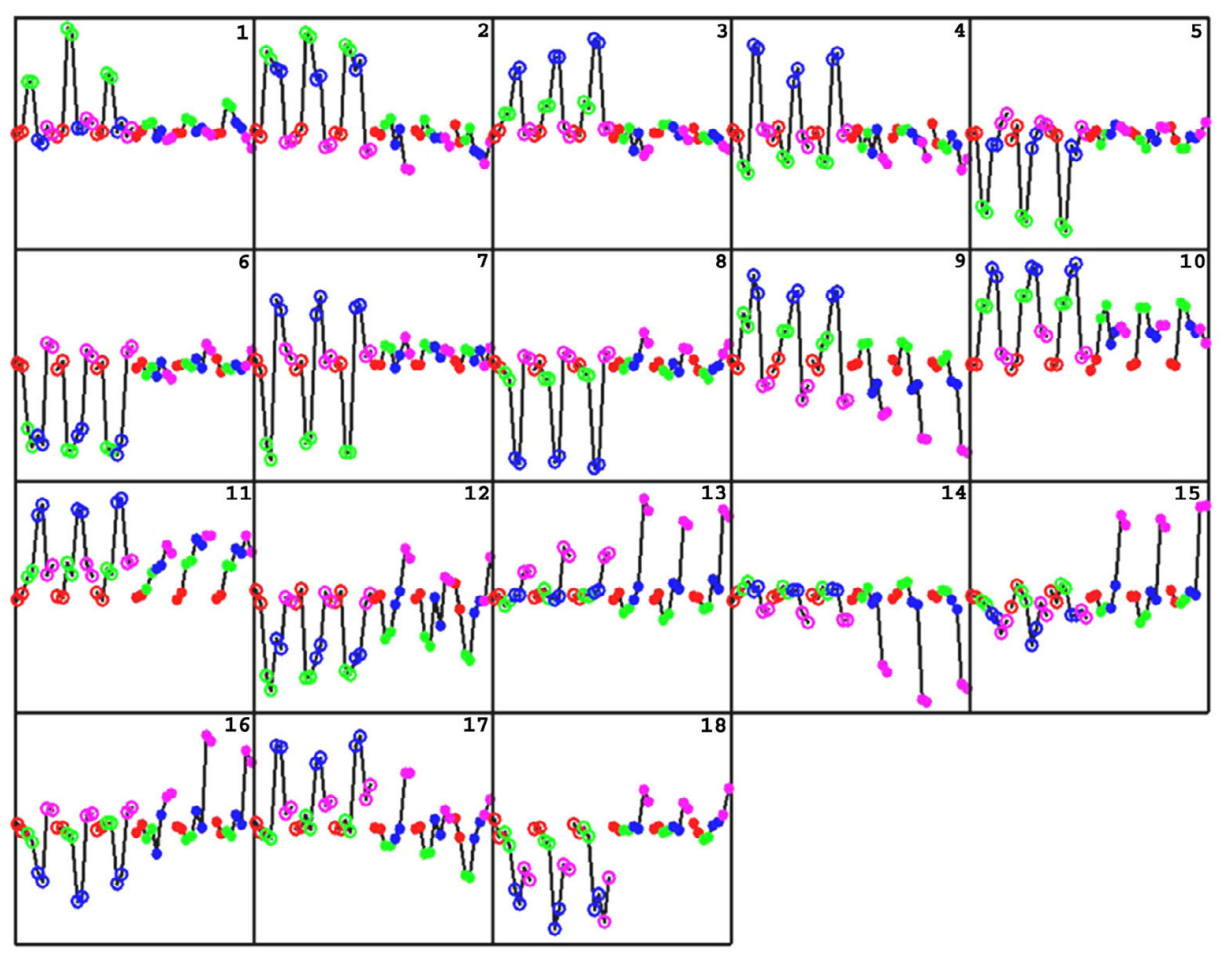
**The patterns extracted by EPIG from the combined UV- and IR- treated data**. In each of these patterns, 1 to 18, the first half with open circles were UV-treated and the second half with solid circles were IR-treated. For each treatment, there were three individual cell lines, F1-HTERT, F3-HTERT and F10-HTERT, positioned from left to right. Each cell line consisted of eight data points with four different treatment conditions, i.e., sham-treatment and 2, 6, and 24 h post-treatment colored red, green, blue and magenta, respectively. The vertical axes with zero at the middle are the changes in gene expression (log2 intensity) relative to the sham-treated controls.

**Figure 5:**
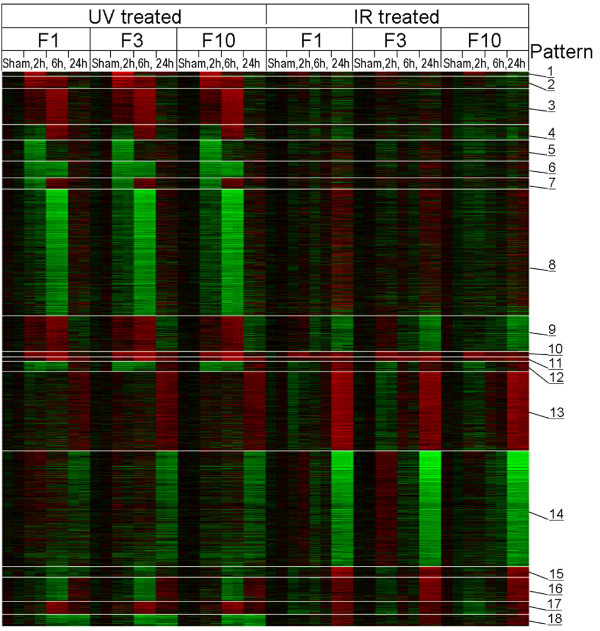
**Heat Map of the 2661 genes selected by EPIG**. From top to bottom are the 2661 genes selected by EPIG listed in an order from Pattern 1 to 18. The left half is UV-treated and the right half is IR-treated. For each treatment, three individual cell lines, F1-HTERT, F3-HTERT and F10-HTERT, are positioned from left to right. Each cell line consisted of four different treatment conditions, sham-treatment, 2, 6, and 24 h post-treatment from left to right. Red and green colors correspond to up and down regulation, respectively, with a darker color denoting less differential expression.

We next applied EPIG to a microarray data set that combined gene expression profiles of both ionizing radiation (IR)- and ultraviolet (UV)-treated human fibroblast cells with two goals in mind: 1) to find similar and dissimilar responses between treatments and 2) to reveal differences in gene regulation upon DNA damage caused by IR or UV. In each of the two treatments, the data consisted of four biological states, i.e. sham-treated, 2 h-, 6 h-, and 24 h post UV- or IR-treatment. A gene expression profile consists of eight inter-groups, corresponding to four states from the two treatments. Each of the intra-groups contains six data points from three biological replicates and two technical replicates (dye-swap pairs) for a given treatment at a given time point. As such, each gene expression profile consisted of 48 data points. EPIG analysis using the whole data as its input resulted in total of 18 patterns as shown in Figure 4 with a total of 2661 co-expressed genes being identified. Each of the co-expressed genes was categorized to a particular pattern. Figure 5 is a heat map of the 2661 genes that are arranged in the order of pattern number from top to bottom. Table 3 lists the number of genes in each of the patterns and denotes their over-represented Gene Ontology biological processes[19].

**Table 3:** Pattern response trends and selected over represented Gene Ontology categories.

Pattern	No. of genes	UV Response trends	IR Response trends	Selected over represented Gene Ontology categories*
1	21	Up regulated at 2 h post UV	Insignificant response	Transcription factor complex, development, nucleoplasm, regulation of transcription from Pol II promoter, morphogenesis
2	57	Up regulated at 2 h and 6 h post UV	Insignificant response	Nucleus, nucleic acid binding
3	173	Moderately up regulated at 2 h and peak 6 h post UV	Insignificant response	RNA metabolism, RNA processing, methyltransferase activity, nucleolus
4	73	Moderately down regulated at 2 h and up regulated at 6 h post UV	Insignificant response	Metabolism, nucleobase\, nucleoside\, nucleotide and nucleic acid metabolism, regulation of transcription, DNA-dependent
5	100	Down regulated at 2 h post UV	Insignificant response	Regulation of transcription, transcription\DNA-dependent, nucleic acid binding, nucleus
6	79	Down regulated at 2 h and 6 h post UV	Insignificant response	Protein serine/threonine kinase activity, nucleus
7	52	Down regulated at 2 h and Moderately up regulated 6 h post UV	Insignificant response	Transcription regulator activity
8	616	Down regulated at 6 h post UV	Insignificant response	Purine nucleotide binding, protein modification, protein amino acid phosphorylation, ubiquitin cycle, kinase activity, cell growth and/or maintenance
9	172	Moderately up regulated at 2 h and peak 6 h post UV	Moderately up regulated at 2 h and down regulated at 24 h post IR	Nucleolus, ribosome biogenesis and assembly, mitotic cell cycle, rRNA processing, DNA replication, S phase of mitotic cell cycle
10	23	Moderately up regulated at 2 h and peak 6 h post UV	Up regulated at 2 h post IR, then decrease at 6 h and remained stable through 24 h post IR.	cell proliferation
11	19	Moderately up regulated at 2 h and peak 6 h post UV	Progressively up regulated from 2 h to 24 h post IR	
12	47	Down regulated at 2 h and Moderately down regulated 6 h post UV	Down regulated at 2 h post IR	protein binding
13	385	Moderately up regulated at 24 h post UV	Up regulated at 24 h post IR	Lysosome, lytic vacuole, complement activation
14	563	Moderately down regulated at 24 h post UV	Down regulated at 24 h post IR	Mitotic cell cycle, DNA replication and chromosome cycle, M phase, nuclear division, cell growth and/or maintenance, RNA processing, RNA metabolism, response to DNA damage stimulus, DNA repair, cell cycle checkpoint, cell growth and/or maintenance, G1/S transition of mitotic cell cycle, G2/M transition of mitotic cell cycle,
15	49	Insignificant response	Up regulated at 24 h post IR	cell adhesion
16	115	Down regulated at 6 h post UV	Up regulated at 24 h post IR	catalytic activity
17	61	Up regulated at 6 h post UV	Moderately down regulated at 2 h post IR	DNA binding
18	56	Down regulated at 6 h and 24 h post UV	Moderately up regulated at 24 h post IR	plasma membrane, morphogenesis

*Includes biological processes, molecular function and cellular component.

Each pattern shown in Figure 4 includes both UV (the first half of the profile from left to right) and IR (the second half) treatments. For each treatment there are three individual cell lines (F1-hTERT, F3-hTERT and F10-hTERT). Each one consists of four time-series points, i.e. sham-treated controls (red), 2 h- (green), 6 h- (blue), and 24 h- (magenta) post UV or IR treatments. Patterns 1 through 8 show UV-specific expression (either up- or down-regulated) with little or no changes in gene expression in IR-treated cells. UV-specific up-regulation of gene expression (Pattern 1 to 4) happened only at early time points, 2 and/or 6 h after UV irradiation, and fully recovered to baseline levels at 24 h. As shown in Table 3, the 324 genes in Patterns 1–4 were related to transcriptional regulation, RNA processing and nucleic acid binding. UV-specific down-regulation of gene expression (Patterns 5 to 8) also occurred at early time points (2 h and/or 6 h post UV). Biological processes related to regulation of transcription were also over-represented by these genes. In addition, over 600 genes in Pattern 8, which were substantially repressed at 6 h post UV, contained about 30 biological processes over-represented including purine nucleotide binding, protein modification, ubiquitin cycle, kinase activity, and cell growth.

Genes in Patterns 9 to 15 responded to both UV- and IR-treatment but with different time dependencies. In Pattern 9 expression was up-regulated at 2 and 6 h post UV-treatment and then recovered to near baseline levels at 24 h. Genes in this pattern were minimally up-regulated at 2 h post IR but showed substantial down-regulation at 6 h and even greater down-regulation at 24 h. Many genes that are maximally expressed in S phase were in this pattern, including CDC6, FEN1, MSH6, ORC6L, PCNA, POLG, RBM14, CCNE2 and TOP3A. The changes in expression of these genes were coincidental with changes in the S phase compartment of the cell cycle. S phase cells were increased over control at 2 and 6 h post-UV (data not shown), but were moderately reduced relative to control at 6 h and markedly reduced at 24 h post-IR [20].

Patterns 10 and 11 contained 42 genes including the p53-target genes GDF15, BTG2, PLK2, BAK1, PLK3 and CDKN1A (Pattern 10) and TP53INP1, SESN1, DDB2 and FDXR (Pattern 11). All of these genes were up-regulated at 2 h after UV treatment, they peaked at 6 h and then returned to baseline at 24 h. However, they responded differently to IR. In Pattern 10, the genes peaked at 2 h post IR then decreased to a stable level at 6 h and 24 h. The genes in Pattern 11 displayed continuous increases in expression until 24 h after IR.

Patterns 13 to 15 contained 997 genes in total that were mainly regulated at the late time point after treatment (24 h). More than 500 genes in Pattern 14 were down-regulated at 24 h post IR or UV treatment. This pattern included many cell cycle-regulated genes functioning in the G1/S and G2/M transitions, such as CDK2, CDC2 and MCM2. These genes were strongly down-regulated in IR-treated cells, but had substantially less change in UV-treated cells. Both Patterns 13 and 15 show late up-regulated responses, but genes in former were up-regulated after treatment with both UV and IR while those in the latter only responded to IR. The top biological process in Pattern 13 was complement activation with lysosome and lytic vacuole being the main cellular components that the products of the genes in the patterns might be associated with or located in. Finally, many genes exhibited co-expression in Patterns 16 (115 genes), 17 (61 genes) and 18 (56 genes) but UV and IR treatments induced opposite changes in their gene expression.

As a comparison, we also applied CLICK to the 2726 genes identified as differentially expressed using the SNR (> 3) and magnitude of expression (> 0.5) criteria. This set of genes included all 2661 co-expressed genes identified by EPIG. In this case, CLICK clustered the gene expression data into 11 groups (in Figure S4 in Additional file 1). Similar patterns from the centroids of the clusters were revealed in comparison to the ones extracted by EPIG. However, CLICK was unable to reveal some patterns extracted by EPIG which we consider to have biological importance related to DNA repair in responding to UV and IR treatments. Among them are EPIG's Patterns 9, 10 and 11 (see Figure 4). As presented above, many of the genes in Pattern 9 were S phase-related and the majority of genes in Patterns 10 and 11 were known to be related to p53-dependent cell cycle control.

### Homogeneity evaluation of the patterns/clusters of gene expression profiles

It is vital that the extracted patterns and their associated genes can be inspected for their biological meaningfulness as presented above. On the other hand, it is also essential that that the extracted patterns or clusters of genes formed be validated objectively using some evaluation indicies. Although there were a number of methods developed to validate clusters, each of them is suitable to some specific applications. For example, one may use the General Silhouette (GS) to measure the stability of the clustering structure [6]. The higher GS score indicates better formed clusters. But the GS measure seems to work best for cases where the number of clusters are small [21,22]. Given the above simulated data, we obtained GS scores of 0.77 and 0.71 for the pattern from EPIG and the clusters from CLICK, respectively. However, when we applied the GS measure to either of the above dauer recovery and L1 starvation or UV and IR DNA damage data sets, the GS scores was deemed inappropriate to judge cluster validity due to the higher numbers of extracted clusters. Other methods, for example, the Gap statistic [22], validate clusters depending on the number of groups in the data as determined a priori. However, both EPIG and CLICK are unsupervised approaches. Therefore, to objectively and appropriately compare the two methods, we calculated the overall average homogeneity [7] within clusters (*i.e. *the pattern-categorized gene expression profiles in the case of EPIG) and the averaged correlations between clusters/extracted patterns. The overall average homogeneity measures the amount of cohesion within a cluster/pattern whereas the averaged correlation measures the amount of separation between clusters/patterns [23]. The results are listed in Table 4. As can be seen, in each of the cases of the three data sets, the overall average homogeneities from EPIG are consistently higher than those from CLICK and CAST (with affinity threshold set to 0.7, 0.8, or 0.85) suggesting that EPIG has better compactness of the profiles in the patterns than the clusters of the genes generated by the other clustering algorithms. The averaged correlations for all the methods were low (<= +/- 0.35), indicating that the expression of the genes in the patterns were quite dissimilar each other. However, it is clear from this result that CLICK performed better than EPIG and CAST with respect to the between cluster correlations (i.e., CLICK had a lower average correlation between clusters/extracted patterns than EPIG and CAST did).

Another observation is that the numbers of clusters generated by CLICK were always lower than the number of patterns extracted by EPIG and the number of clusters generated by CAST was about 8 (on average and not including singletons) when different affinity threshold values were used for analysis. However, if we use the whole data set as the input data for CLICK, as what was done for the analysis of the data with EPIG, CLICK produces over 60 clusters in both dauer recovery/L1 starvation and UV/IR DNA damage data sets. A Figure of merit (FOM) [24] analysis of the either data set showed that for cases when the number of clusters were larger than 20, the adjusted FOM values slowly decreased. This suggests that the number of clusters of genes in the data set could be between 5 and 20, well below the 60 clusters produced by CLICK when using the entire gene expression data set (Figure S5 in Additional file 1).

## Discussion

With a pair-wise calculation of the Pearson's correlation coefficient *r*, co-expressed profiles form discrete local clusters or mountains in a correlation topomap [18]. The probability is less than 10^-12 ^that two arbitrary and independently generated data sets of size 48, e.g. the joined UV and IR data set shown in Figure 4, are correlated with an *r*-value greater than 0.8 [25]. When there exist tens of thousands of gene expression profiles, many of them, which are inconsistently expressed among replicate groups or intra-groups, may appear to be similar by chance and form a correlation local cluster due to stochastic noise. Unlike factor analysis methods, such as ICA, or clustering methods, such as CLICK, *K*-means or SOM, our approach called Extracting Patterns and Identifying co-expressed Genes (EPIG) not only calculates the similarity among the profiles, but also evaluates each profile via signal-to-noise (S/N) ratio measurements (Equations 1 and 3). Through a filtering procedure, EPIG removes profiles that don't fit into a pattern. Only the profiles with high S/N ratios and desirable magnitudes of expression change are included in the formation of patterns representing co-expressed genes. With such a profile evaluation strategy, EPIG is able to extract patterns of co-expressed genes without predefined seeding.

In a head-to-head comparison, EPIG competed with CLICK and CAST in the analysis of a simulated data set by 1) extracting all of the designated patterns, 2) accurately categorizing the profiles to their appropriate patterns, and 3) generating patterns of profiles with higher homogeneity and more stability (Table 4). However, it is clear that CLICK outperformed EPIG and CAST in terms of generating clusters/extracted patterns that are more dissimilar to each other (i.e., they have a lower average correlation between clusters/patterns). Furthermore, given the two experimental data sets presented above (one from the public domain), EPIG extracted more patterns of gene expression than CLICK (Tables 2 and 4). The patterns extracted by EPIG which were not represented by any of the cluster centroids generated by CLICK contained genes which related to key biological responses coupled to the experimental treatments. For example, in the case of UV and IR DNA damage, the patterns extracted by EPIG contained p53 cell cycle control target genes (in Patterns 10 and 11) and many S phase genes (in Pattern 9) of the mitotic cell cycle (Figure 4).

**Table 4:** Homogeneity of gene expression profiles within patterns and correlations between patterns

Data	Algorithm	Number of patterns	Overall average homogeneity within patterns	Averaged correlation between patterns
The simulated data	EPIG	5	0.95	0.29
	CLICK	3	0.72	-0.35
	CAST*	8	0.30	-0.08
Dauer recovery and L1 starvation data	EPIG	18	0.83	-0.03
	CLICK	5	0.74	-0.15
UV and IR DNA damage data	EPIG	18	0.84	-0.02
	CLICK	11	0.78	-0.08

* Results generated with an affinity threshold value of 0.8. Two singleton profiles were not reported or included in the analysis of the homogeneity and correlation measures.

**Figure 6:**
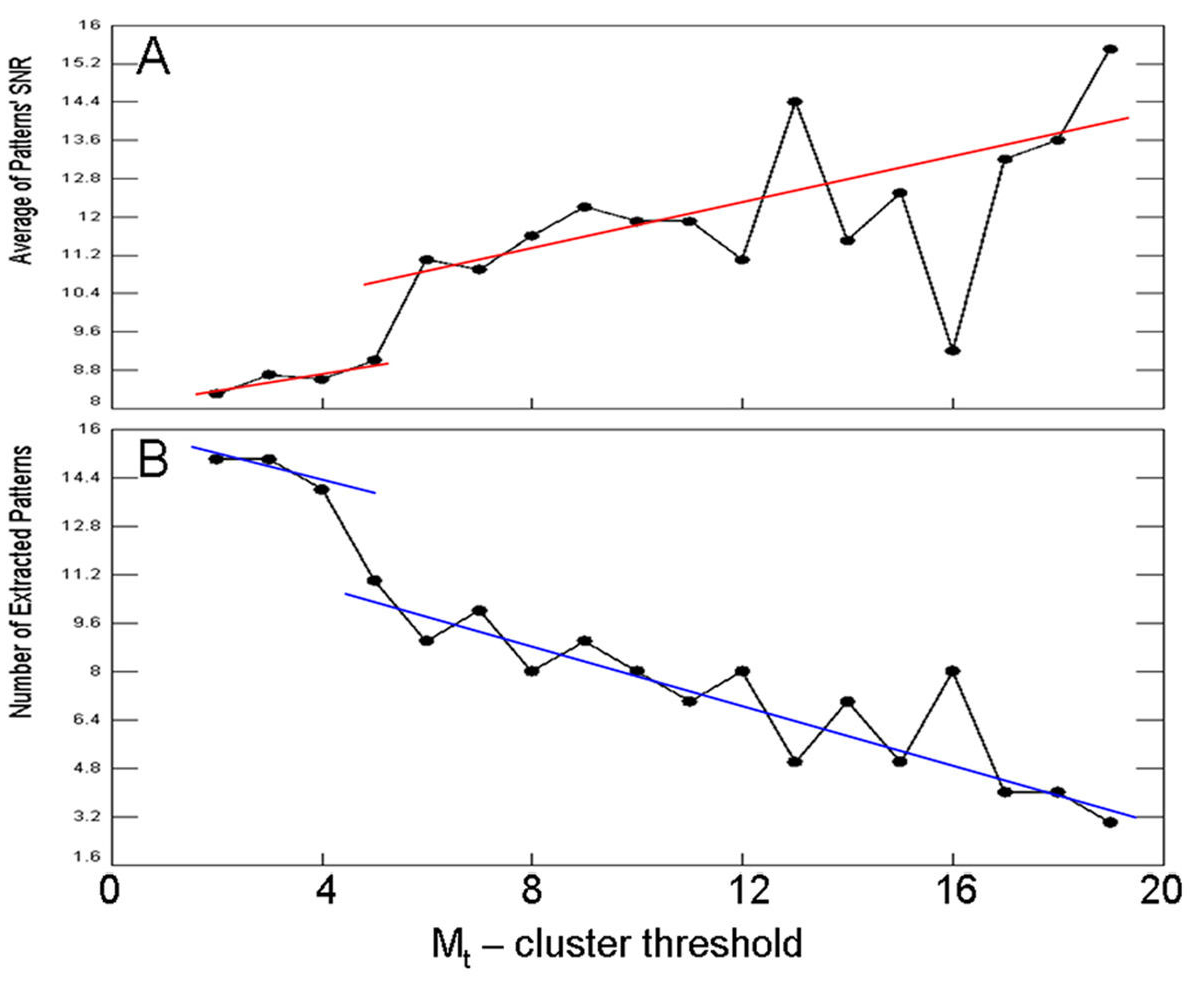
**Optimization of the M_t _value**. Cluster size threshold M_t _(the horizontal axis) verses average of patterns' SNR (A) and number of extracted patterns (B).

There are two main thresholds used in EPIG pattern extraction: the local cluster size threshold M_*t *_and the correlation threshold R_*t*_. R_*t *_determines the closeness in similarity that is allowed among the extracted patterns. Depending on the sample size, one may determine R_*t *_such that the most similar patterns possess clear response differences. For example, in Figure 4, Patterns 5 and 6 have a correlation *r*-value of 0.77. But the two contain genes with expression patterns that display a clear difference in the response to UV-induced DNA damage. In Pattern 5, gene expression was repressed only at 2 h post-UV while in Pattern 6, gene expression was repressed at both 2 and 6 h post-UV. M_*t *_is the minimum number of the genes in a local cluster needed to have a profile candidate deemed as a pattern. The value of M_*t *_affects the pattern extraction outcome. Higher M_*t *_values may cause a meaningful pattern with a lower number of co-expressed genes to be concealed. On the other hand, lower M_*t *_values may lead to the extraction of some patterns lacking biological meaningfulness. To test for an optimal M_*t *_setting, we varied its values from 2 to 19 and performed EPIG analysis on the IR-treated gene expression data. Figure 6 shows that the average Pattern SNR increased with the increase of M_*t*_, while the number of extracted patterns decreased. This result is seems plausible considering the observation that as M_*t *_increases, more correlation local clusters are filtered-out since their cluster sizes are less than M_*t*_. The fewer extracted patterns then have higher averaged SNRs. However, when M_*t *_≥ 6, the SNR had an up-shift and the number of extracted patterns had a down-shift. This result prompted us to set M_*t *_to 6 in the given data set. To be precise, one should vary these thresholds empirically for a given data set to examine the outcomes. We have done just that and have concluded that, upon many sets of the gene expression data analyzed by using EPIG, selections of M_*t *_at 6 and R_*t *_at 0.8 have worked reasonably well (data not shown). Incidentally, there may be some genes with profiles not similar to any other gene(s) or their related local cluster had a size less than M_*t*_. Then these "orphan" genes (singletons) will not be considered as a pattern candidate nor will they be categorized to any extracted patterns. Attention certainly needs to be paid to these orphan genes, as a part of the EPIG analysis result, to determine if they have a unique role in the treatment response.

EPIG is a general method for gene expression analysis when the data consists of profiles with multiple inter-groups and multiple samples intra-groups. Each intra-group has a specific biologically relevant factor. The inter groups account for the factor variations. For example, in the IR time- series data set, since the intra-group included both biological and technical replicates, the common response features among different cell lines were identified. On the other hand, if the intra-group included only the technical replicates, then one would reasonably expect to extract patterns representing idiosyncratic responses in individual cell lines. The responses to DNA damage that are common among biological individuals are intriguing because they are conserved, but individual-specific responses also are of interest as they point to inter-individual variations in response to external perturbations.

The application to the joined IR and UV data set had eight inter-groups, four each (i.e. sham, 2 h, 6 h and 24 h post-treatment) to IR and UV respectively. In this case, similar and dissimilar responses between IR- and UV- induced DNA damage can be clearly observed (Figure 4). For example, UV-specific response patterns included genes functioning in transcription regulation, RNA processing, nucleotide binding, and cell growth (Patterns 1 through 8 in Figure 4 and Table 3). The over-represented categories of Gene Ontology from the 616 genes in Pattern 8 included purine nucleotide binding, protein modification, ubiquitin cycle, kinase activity, and cell growth. It appears that protein kinases may be generally down-regulated specifically via phosphorylation in response to UV-induced DNA damage [26,27]. Two early response Patterns 10 and 11 in Figure 4 showed that both UV and IR caused these genes to be up-regulated, but in different ways. Many of the genes in these two patterns have been widely studied and known to be related to p53-dependent cell cycle control. The two different patterns of response to IR imply that factors other than p53 also influence the expression of p53-target genes. Pattern 14 in Figure 4 showed similar late down-regulation responses to both UV and IR treatments. There were 563 genes in Pattern 14 participating in a number of important biological processes among them were mitotic cell cycle, DNA replication, DNA repair, cell cycle checkpoint, and G_0_-like status transition [20].

In general, the inter-group-related factors are not limited to the time variable only. As a matter of fact, EPIG has been applied to many different data sets, where the variable factors include time, treatments (such as chemicals, radiation, knock-out), doses, organs (such as blood, liver, kidney), or organ sections (such as left or right lobe in liver). As such, EPIG is a robust, flexible and new pattern extraction method which is generally applicable to a variety of microarray data sets.

## Conclusion

EPIG competed with CLICK and performed better than CAST in extracting patterns from simulated data. However, EPIG extracted more biological informative patterns and co-expressed genes from real biological data: both C. elegans and IR/UV-treated human fibroblasts. Using Gene Ontology analysis of the genes in the patterns extracted by EPIG, several key biological categories related to p53-dependent cell cycle control were revealed from the IR/UV data. Among them were mitotic cell cycle, DNA replication, DNA repair, cell cycle checkpoint, and G_0_-like status transition. The extraction of these biologically responsive processes by EPIG provides a deeper understanding of the underlying biological mechanism(s) in the perturbed system.

## Methods

### Simulated data

A data set comprised of numeric data with 90 profiles and 24 objects (four inter- groups *i *(*i *= 1,...,4) and six intra-groups *j *(*j *= 1,...,6)) was simulated from six distinct probability distributions. Normal deviates were drawn at random to generate 15 profiles for each of the six distributions. Table 1 lists the mean value distributions used in the simulation of the data where the standard deviation was set to be constant at 0.4.

### Microarray data

Microarray gene expression data was acquired from three telomerized normal human fibroblast lines (logarithmically growing), F1-hTERT, F3-hTERT and F10-hTERT[28,29] that were sham-treated or treated with 1.5 Gy ionizing radiation (IR) or 6 J/m^2 ^ultraviolet (UV [from a 254 nm radiation source]) and harvested at 2, 6 or 24 h after the treatment. Briefly, total RNA isolated from cells harvested at each time point was subjected to microarray analysis by using Agilent 22,000 element human 1A arrays. The labelled cRNA from sham- or radiation-treated samples was hybridized to the microarray along with a labelled global reference cRNA. Hybridization of sample RNA against reference RNA was performed with a dye swap (cytofluor reversal).

### Microarray data pre-processing

The extracted intensity data were pre-processed by array-based Systematic Variation Normalization (SVN) [30], profile-based dye-swap correction to remove dye labelling affects, and to align biological reference states. The latter was done in such a way that the sham-treated samples were used as a reference state and the averaged sham-treated state of the dye-swapped pair was aligned to zero as a baseline. All other treated states were then adjusted by the same amount accordingly. It is plausible that each of the three human cell lines used for analysis may not have the same sham state. The alignment of the sham states aims at eliminating the biological variability and focuses the analysis on the relative changes upon IR or UV treatments. In this case, the 2-dimensional matrix of compiled gene expression data consisted of 16,757 rows of unique genes (grouped by UniGene) and 48 columns consisting of, for each of the three cell lines, dye-swapped replicates of IR-sham-treatment and 2 h, 6 h and 24 h after IR, UV-sham-treatment and 2 h, 6 h and 24 h after UV.

### EPIG

A compiled microarray gene expression data set (in this study, as conventionally presented, the log_2 _pixel intensity ratio values) consists of a 2-dimensional matrix, in which each row represents a gene expression profile and each column represents an array. Upon sample perturbation or variation in biological factors, such as agent, dose, time or tissue, a gene expression profile can be made up of inter-group and intra-group samples. The arrays in an intra-group sample have a factor in common, *e.g. *biological replicates. The arrays in inter-group samples possess different factors, e.g., sham-treatment and time points post-UV or IR treatment. We denote each datum of log_2 _ratio as g_*ij *_in a gene expression profile, where *i *refers to a inter-group index from 1 to *m*, *j *is the intra-group index from 1 to n_*i*_, m is the number of inter-groups and n_*i *_is the number of arrays in *i*^th ^inter-group. To evaluate such a profile, we calculate each intra-group average g¯i
 MathType@MTEF@5@5@+=feaafiart1ev1aaatCvAUfKttLearuWrP9MDH5MBPbIqV92AaeXatLxBI9gBaebbnrfifHhDYfgasaacPC6xNi=xH8viVGI8Gi=hEeeu0xXdbba9frFj0xb9qqpG0dXdb9aspeI8k8fiI+fsY=rqGqVepae9pg0db9vqaiVgFr0xfr=xfr=xc9adbaqaaeGacaGaaiaabeqaaeqabiWaaaGcbaGafm4zaCMbaebadaWgaaWcbaGaemyAaKgabeaaaaa@2EC9@ and sample variance si2
 MathType@MTEF@5@5@+=feaafiart1ev1aaatCvAUfKttLearuWrP9MDH5MBPbIqV92AaeXatLxBI9gBaebbnrfifHhDYfgasaacPC6xNi=xH8viVGI8Gi=hEeeu0xXdbba9frFj0xb9qqpG0dXdb9aspeI8k8fiI+fsY=rqGqVepae9pg0db9vqaiVgFr0xfr=xfr=xc9adbaqaaeGacaGaaiaabeqaaeqabiWaaaGcbaGaem4Cam3aa0baaSqaaiabdMgaPbqaaiabikdaYaaaaaa@2FBC@. We define a gene expression profile's signal as

S={max⁡{g¯i},ifmin⁡{g¯i}>0−min⁡{g¯i},elseifmax⁡{g¯i}<0max⁡{g¯i}−min⁡{g¯i}otherwise.
 MathType@MTEF@5@5@+=feaafiart1ev1aaatCvAUfKttLearuWrP9MDH5MBPbIqV92AaeXatLxBI9gBaebbnrfifHhDYfgasaacPC6xNi=xI8qiVKYPFjYdHaVhbbf9v8qqaqFr0xc9vqFj0dXdbba91qpepeI8k8fiI+fsY=rqGqVepae9pg0db9vqaiVgFr0xfr=xfr=xc9adbaqaaeGacaGaaiaabeqaaeqabiWaaaGcbaGaem4uamLaeyypa0ZaaiqaaeaafaqaaeWacaaabaGagiyBa0MaeiyyaeMaeiiEaG3aaiWaaeaacuWGNbWzgaqeamaaBaaaleaacqWGPbqAaeqaaaGccaGL7bGaayzFaaGaeiilaWIaemyAaKMaemOzayMagiyBa0MaeiyAaKMaeiOBa42aaiWaaeaacuWGNbWzgaqeamaaBaaaleaacqWGPbqAaeqaaaGccaGL7bGaayzFaaaabaGaeyOpa4JaeGimaadabaGaeyOeI0IagiyBa0MaeiyAaKMaeiOBa42aaiWaaeaacuWGNbWzgaqeamaaBaaaleaacqWGPbqAaeqaaaGccaGL7bGaayzFaaGaeiilaWIaemyzauMaemiBaWMaem4CamNaemyzauMaemyAaKMaemOzayMagiyBa0MaeiyyaeMaeiiEaG3aaiWaaeaacuWGNbWzgaqeamaaBaaaleaacqWGPbqAaeqaaaGccaGL7bGaayzFaaaabaGaeyipaWJaeGimaadabaGagiyBa0MaeiyyaeMaeiiEaG3aaiWaaeaacuWGNbWzgaqeamaaBaaaleaacqWGPbqAaeqaaaGccaGL7bGaayzFaaGaeyOeI0IagiyBa0MaeiyAaKMaeiOBa42aaiWaaeaacuWGNbWzgaqeamaaBaaaleaacqWGPbqAaeqaaaGccaGL7bGaayzFaaaabaGaem4Ba8MaemiDaqNaemiAaGMaemyzauMaemOCaiNaem4DaCNaemyAaKMaem4CamNaemyzaugaaaGaay5EaaGaeiOla4caaa@8741@

where 1 ≤ *i *≤ *m*. We define a profile's noise estimate as the square-root of the pooled variance, i.e.

N=∑im[(ni−1)⋅si2]∑im(ni−1)∑im1ni
 MathType@MTEF@5@5@+=feaafiart1ev1aaatCvAUfKttLearuWrP9MDH5MBPbIqV92AaeXatLxBI9gBaebbnrfifHhDYfgasaacPC6xNi=xI8qiVKYPFjYdHaVhbbf9v8qqaqFr0xc9vqFj0dXdbba91qpepeI8k8fiI+fsY=rqGqVepae9pg0db9vqaiVgFr0xfr=xfr=xc9adbaqaaeGacaGaaiaabeqaaeqabiWaaaGcbaGaemOta4Kaeyypa0ZaaOaaaeaajuaGdaWcaaqaamaaqahabaWaamWaaeaacqGGOaakcqWGUbGBdaWgaaqaaiabdMgaPbqabaGaeyOeI0IaeGymaeJaeiykaKIaeyyXICTaem4Cam3aa0baaeaacqWGPbqAaeaacqaIYaGmaaaacaGLBbGaayzxaaaabaGaemyAaKgabaGaemyBa0gacqGHris5aaqaamaaqahabaGaeiikaGIaemOBa42aaSbaaeaacqWGPbqAaeqaaiabgkHiTiabigdaXiabcMcaPaqaaiabdMgaPbqaaiabd2gaTbGaeyyeIuoaaaGcdaaeWbqaamaalaaabaGaeGymaedabaGaemOBa42aaSbaaSqaaiabdMgaPbqabaaaaaqaaiabdMgaPbqaaiabd2gaTbqdcqGHris5aaWcbeaaaaa@56BA@

where the sample variance

si2=∑jni(gij−g¯i)2ni−1.
 MathType@MTEF@5@5@+=feaafiart1ev1aaatCvAUfKttLearuWrP9MDH5MBPbIqV92AaeXatLxBI9gBaebbnrfifHhDYfgasaacPC6xNi=xI8qiVKYPFjYdHaVhbbf9v8qqaqFr0xc9vqFj0dXdbba91qpepeI8k8fiI+fsY=rqGqVepae9pg0db9vqaiVgFr0xfr=xfr=xc9adbaqaaeGacaGaaiaabeqaaeqabiWaaaGcbaGaem4Cam3aa0baaSqaaiabdMgaPbqaaiabikdaYaaakiabg2da9KqbaoaalaaabaWaaabCaeaacqGGOaakcqWGNbWzdaWgaaqaaiabdMgaPjabdQgaQbqabaGaeyOeI0Iafm4zaCMbaebadaWgaaqaaiabdMgaPbqabaGaeiykaKYaaWbaaeqabaGaeGOmaidaaaqaaiabdQgaQbqaaiabd6gaUnaaBaaabaGaemyAaKgabeaaaiabggHiLdaabaGaemOBa42aaSbaaeaacqWGPbqAaeqaaiabgkHiTiabigdaXaaakiabc6caUaaa@489A@

From Equations 1 and 2, we define a profile's signal-to-noise ratio as

SNR=SN
 MathType@MTEF@5@5@+=feaafiart1ev1aaatCvAUfKttLearuWrP9MDH5MBPbIqV92AaeXatLxBI9gBaebbnrfifHhDYfgasaacPC6xNi=xI8qiVKYPFjYdHaVhbbf9v8qqaqFr0xc9vqFj0dXdbba91qpepeI8k8fiI+fsY=rqGqVepae9pg0db9vqaiVgFr0xfr=xfr=xc9adbaqaaeGacaGaaiaabeqaaeqabiWaaaGcbaGaem4uamLaemOta4KaemOuaiLaeyypa0tcfa4aaSGaaeaacqWGtbWuaeaacqWGobGtaaaaaa@339C@

As can be seen, when *m *= 1, Equation 3 is equivalent to a two sample *t*-test, since by default the log_2 _pixel intensity ratio is the treated against its control. Equation 3 includes the case for *m *> 1, i.e. multiple inter-groups.

In extracting gene expression patterns, EPIG uses a filtering process where all profiles initially are considered as pattern candidates. The pseudo code for the algorithm can be found in the Appendix. Briefly, using all pair-wise correlations, any candidate profile, whose local cluster size is less than a predefined size M_*t *_or its correlation with another profile is higher (> R_*t*_) but has a lower local cluster size M, is removed from pattern construction consideration. Among the remaining profiles, EPIG then creates representative profiles for the corresponding local clusters and removes those profiles with a SNR in Equation 3 less than 3 or magnitude S in Equation 1 less than 0.5. After this filtering processing, the remaining profiles consist of the extracted patterns, which are used to be the representatives to each of the local clusters. Each of the patterns has the highest local cluster size in comparison with other highly similar profiles (e.g. correlation larger than 0.8) in the same local cluster.

Subsequently, EPIG categorizes each gene to the pattern, for which it has the highest correlation with the gene profile. A gene not assigned to any extracted patterns is considered an "orphan" if its highest correlation *r*-value is lower that a given threshold R_*c*_. Typically R_*c *_is set to a value which corresponds to a correlation *p*-value of 10^-4 ^to assure the significance of the co-expression. A Java-based software tool and the source code for EPIG are publicly available [31].

### CLICK

CLuster Identification via Connectivity Kernels (CLICK) analysis of the gene expression data was performed using version 2 of the EXpression ANalyzer and DisplayER (EXPANDER) analysis and visualization tool [7]. The default settings for CLICK were used for all analyses.

### CAST

The Cluster Affinity Search Technique (CAST) for clustering data uses average similarity (affinity) between gene expression patterns and cluster cores (the current ones in the recursive portion of the algorithm) and then adds (and removes) elements from the current core one at a time [5]. An affinity threshold is used to specify the cluster quality – it influences the number and the size of the clusters that are produced. The CAST implementation used for analysis of the data in this paper was based on a Java applet source code [32].

## Appendix

EPIG algorithm

Set thresholds R_*t *_to 0.8, M_*t *_to 6, SNR_*t *_to 3, and S_*t *_to 0.5

FOR each profile *i*

   M_*i *_= 0

   FOR each profile *j*, *j *<*i*

      M_*j *_= 0

      IF correlation *r*-value r_*ij *_> R_*t *_THEN M_*i*++_, M_*j*++_

   END FOR

END FOR

REMOVE profile *i *IF M_*i *_< M_*t*_

SORTING profiles DESCENDING ACCORDING TO M_*i*_

FOR each profile *i*

   SORTING profiles j DESCENDING ACCORDING TO r_*ij*_

   REMOVE profile *j *IF r_*ij *_> R_*t*_

END FOR

FOR each remaining profile *i*

   REPLACE profile *i *WITH a profile which is an average of its top 5 profiles (out of all profiles) having the highest r value

   REMOVE profile *i *IF its SNR < SNR_*t *_OR S < S_*t*_

END FOR

RETURN remaining profiles as the extracted patterns

## Authors' contributions

JWC conceived of the concept of the research and methodology for analysis, performed the analyses of the data, developed the algorithm and wrote part of the manuscript. PRB provided the valuable suggestions for the concept of the research, for all aspects of the methodology and also wrote part of the manuscript. TZ and WKK provided the UV/IR data and offered helpful suggestions for the biological themes related to the extracted patterns. RSP provided useful suggestions related to the utility of the EPIG algorithm and software. All authors read and approved of the final manuscript.

## Supplementary Material

Additional file 1 **Supplemental materials**. Additional data 1.pdf is a pdf file to be opened and viewed with Adobe Acrobat. Click here for file
