# Supplementary material for: Extracting gene expression patterns and identifying co-expressed genes from microarray data reveals biologically responsive processes
Source: BMC Bioinformatics. 2007 Nov 2;8:427. doi: 10.1186/1471-2105-8-427 (PMC2194742; doi:10.1186/1471-2105-8-427)
Supplement: Additional file 1 — Supplemental materials. Additional data 1.pdf is a pdf file to be opened and viewed with Adobe Acrobat. [file 1471-2105-8-427-S1.pdf]

Figure S1. Dauer recovery and L1 starvation gene expression patterns extracted by EPIG

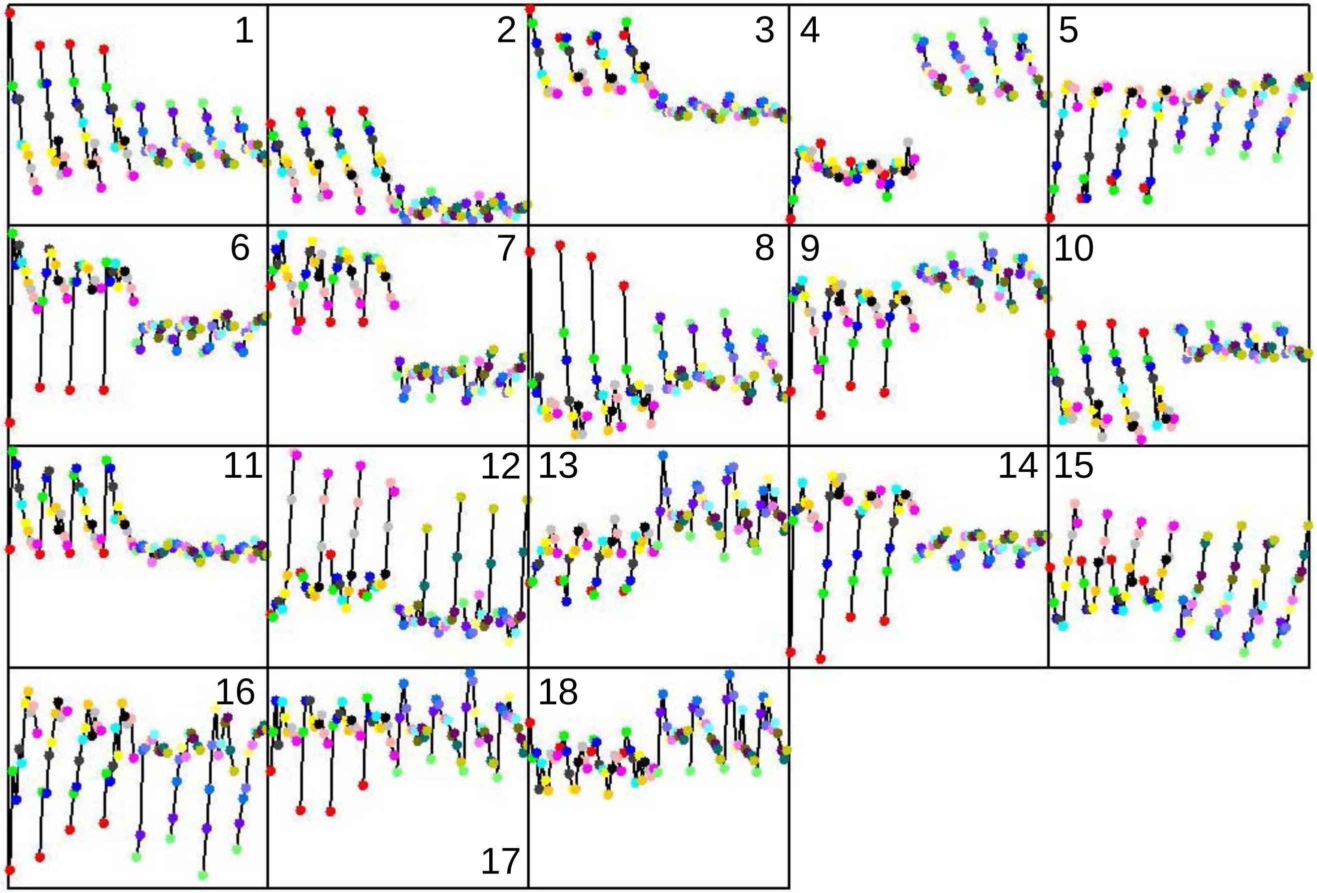


In each pattern extracted by EPIG, the vertical axis is the log2 ratio. The first four curves are biological replicates of dauer recovery time courses marked in different colors from 0 (red dots) to 12 h (magenta dots). The second four curves are biological replicates of L1 starvation time courses from 0 (green dots) to 12 h (olive dots).

Figure S2. Heat Map of 1597 selected genes from EPIG pattern 1 to 18 of dauer recovery and L1 starvation gene expression.  The red color denotes the gene expression up-regulated and the green color down-regulated.

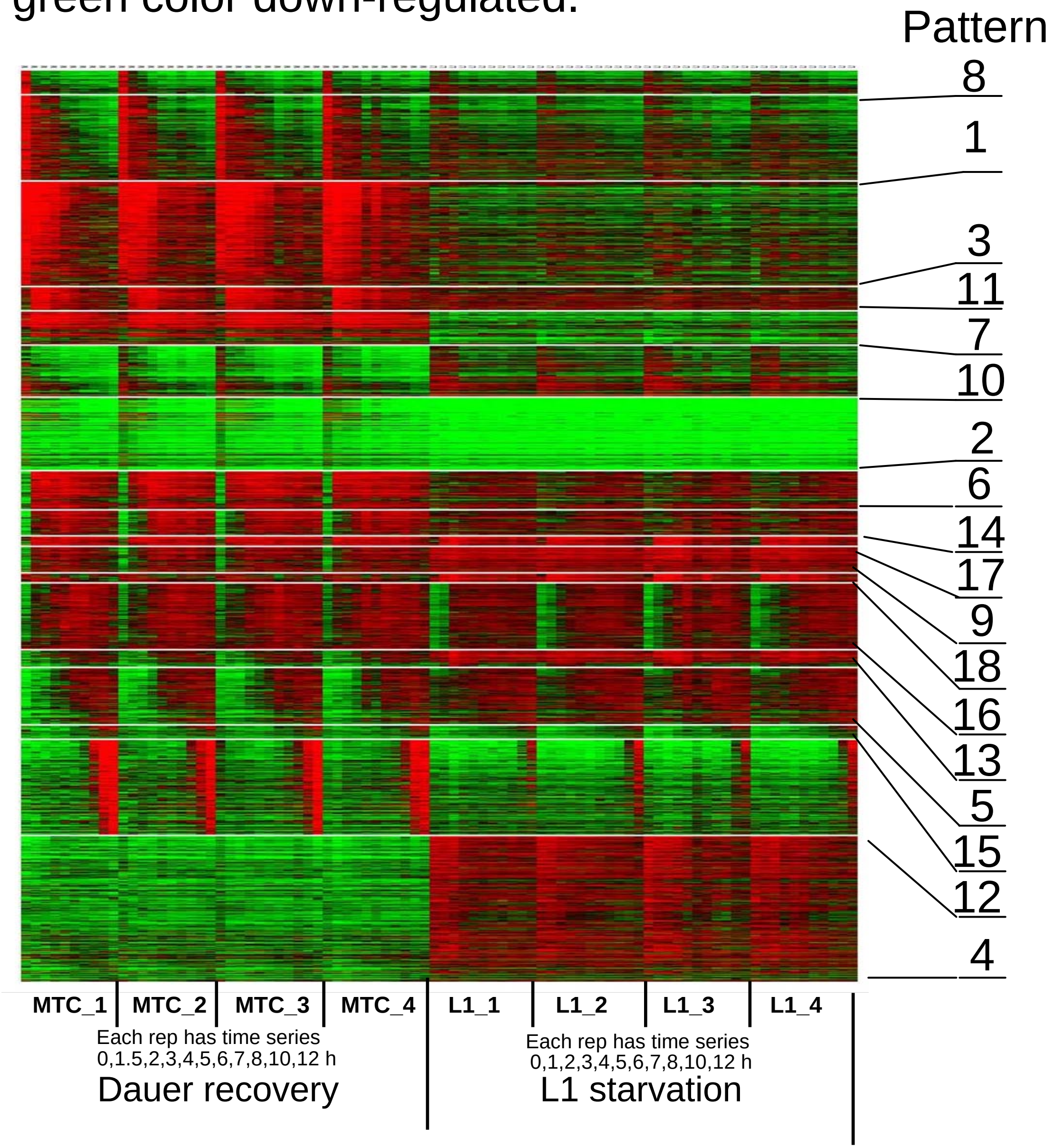

Figure S3. Dauer recovery and L1 starvation gene expression patterns extracted by CLICK

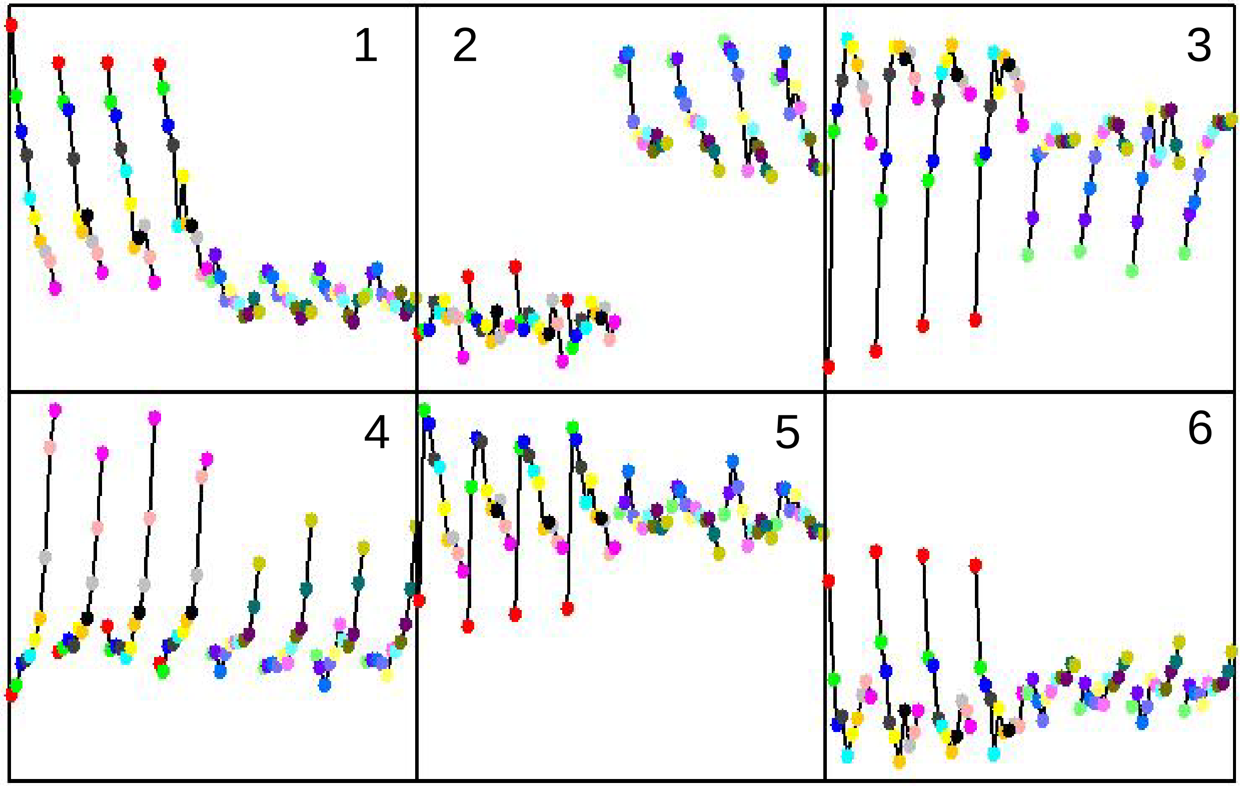


In each pattern extracted by CLICK, the vertical axis is the log2 ratio. The first four curves are biological replicates of dauer recovery time courses marked in different colors from 0 (red dots) to 12 h (magenta dots). The second four curves are biological replicates of L1 starvation time courses from 0 (green dots) to 12 h (olive dots).

Figure S4. UV and IR DNA damage gene expression patterns extracted by CLICK

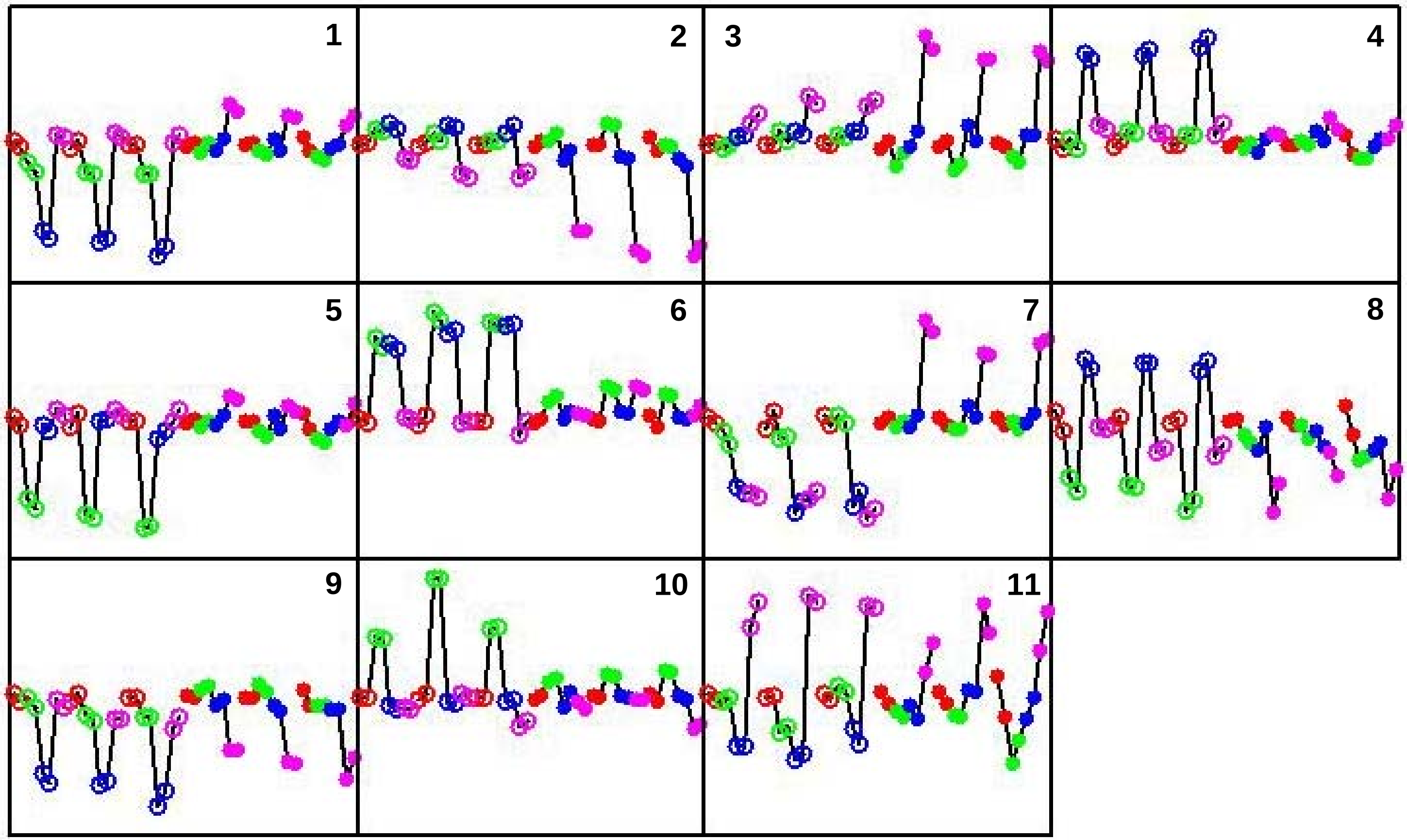


In each pattern, the vertical axis is the log2 ratio. The first three curves are biological replicates of UV treated time series from 0 (red), 2 (green), 6 (blue) to 24 h (magenta). The second three curves are Biological replicates of IR treated time series from 0 (red), 2 (green), 6 (blue) to 24 h (magenta).

Figure S5. FOM cluster validation analysis.
The adjusted FOM decreases sharply when the number of clusters is lower and then decreases slowly when number of clusters is larger than 20.

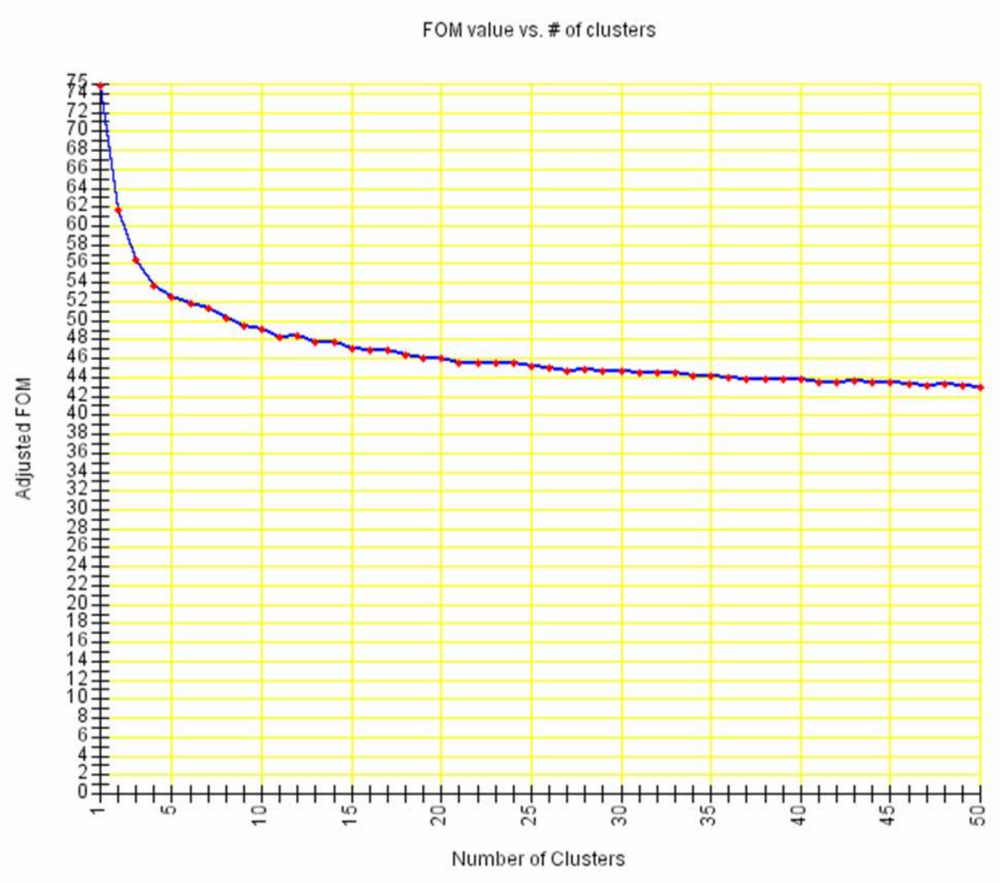

**Table S1. EPIG application of dauer recovery and L1 starvation gene expression in *Caenorhabditis elegans*.**

| EPIG Pattern number | No. of genes | dauer recovery response trends | L1 starvation response trends | Response comparisons |
|---|---|---|---|---|
| 1 | 153 | Significantly up regulated at start, gradually decrease to significantly down regulated at end | No change at start, gradually decrease, to down regulated at end | Similar responses |
| 2 | 130 | No change at start, gradually decrease, to significantly down regulated at end | Down regulated at all time points | Dauer specific response |
| 3 | 188 | Up regulated at start, gradually decrease to no change at end | No change at all time points | Dauer specific response |
| 4 | 263 | Down regulated at all time points | Up regulated at start, gradually decrease to no change at end | Dauer specific response |
| 5 | 101 | Significantly down regulated at start, gradually increase, to moderate up regulated at 8 h and minimally back to no change at end | moderate down regulated at start, gradually increase, to moderate up regulated after 8 h | Similar responses |
| 6 | 68 | Significantly down regulated at start, jump to significantly up regulated after the start, gradually decrease to moderate up regulated at end | No significant change at all time points | Dauer specific response |
| 7 | 59 | moderate up regulated at start, gradually increase, peaked at middle, then back to moderate up at end | moderate down regulated at all time points | Dauer specific response |
| 8 | 42 | Strong up regulated at start, sharply decrease early, stay down regulated late | No change at start, decrease early, stay down regulated late | Similar responses |
| 9 | 45 | moderate down regulated at start, gradually increase, peaked at middle, then back to minimal change at end | Up regulated at all time points | Dauer specific response |
| 10 | 91 | No change at start, gradually decrease, to significantly down regulated at end | No change at start, then, stay minimally down regulated | Dauer specific response |
| 11 | 41 | No change at start, jump to significantly up regulated after the start, gradually decrease to no change at end | No change at all time points | Dauer specific response |
| 12 | 171 | Significantly down regulated at start, sharply increase late, and significantly up regulated at end | Significantly down regulated at start, sharply increase late, and significantly up regulated at end | Similar responses |
| 13 | 29 | moderate down regulated at start, gradually increase to up regulated, peaked at middle, then moderate back to no change at end | No change at start, jump to up regulated early, gradually decrease to minimal change at end | Similar responses |
| 14 | 44 | Significantly down regulated at start, gradually increase to up regulated, peaked at middle, then moderate back at end | No significant change at all time points | Dauer specific response |
| 15 | 23 | No change at start, decrease to down regulated early, then increase to moderate up regulated at end | Significantly down regulated at start, gradually increase to no change at end | Similar responses |
| 16 | 118 | Significantly down regulated at start, gradually increase, to moderate up regulated at 8 h and minimally back to no change at end | Significantly down regulated at start, gradually increase, to moderate up regulated at 8 h and minimally back to no change at end | Similar responses |
| 17 | 16 | Minimal change at start, jump to up regulated, gradually decrease to moderate up regulated at end | Minimal change at start, jump to up regulated, gradually decrease to moderate up regulated at end | Similar responses |
| 18 | 15 | Minimally change at all time points | Minimal change at start, jump to up regulated, gradually decrease to moderate up regulated at end | Similar responses |
